# Associations between circulating cardiovascular disease risk factors and cognitive performance in cognitively healthy older adults from the NuAge study

**DOI:** 10.3389/fnagi.2023.1274794

**Published:** 2023-11-10

**Authors:** Noah D. Koblinsky, Pierre-Hugues Carmichael, Sylvie Belleville, Alexandra J. Fiocco, Pierrette Gaudreau, Carol E. Greenwood, Marie-Jeanne Kergoat, José A. Morais, Nancy Presse, Danielle Laurin, Guylaine Ferland

**Affiliations:** ^1^Rotman Research Institute, Baycrest Health Sciences, Toronto, ON, Canada; ^2^Center d’excellence sur le Vieillissement de Québec, Center de Recherche du Center Hospitalier Universitaire (CHU) de Québec-Université Laval and VITAM-Center de Recherche en Santé Durable, Center Intégré Universitaire de Santé et de Services Sociaux (CIUSSS) de la Capitale Nationale, Québec, QC, Canada; ^3^Center de Recherche de l’Institut Universitaire de Gériatrie de Montréal, CIUSSS du Center-Sud-de-l’Île-de-Montréal, Montreal, QC, Canada; ^4^Department of Psychology, Toronto Metropolitan University, Toronto, ON, Canada; ^5^Center de Recherche du Center Hospitalier de l’Université de Montréal and Faculté de Médecine, Université de Montréal, Montreal, QC, Canada; ^6^Department of Nutritional Sciences, Faculty of Medicine, University of Toronto, Toronto, ON, Canada; ^7^Département de Médecine, Université de Montréal, Montreal, QC, Canada; ^8^Division of Geriatrics, McGill University, Montreal, QC, Canada; ^9^Faculté de Médecine et des Sciences de la Santé de l’Université de Sherbrooke, Sherbrooke, QC, Canada; ^10^Center de Recherche sur le Vieillissement du CIUSSS de l’Estrie-Center Hospitalier Universitaire de Sherbrooke (CHUS), Sherbrooke, QC, Canada; ^11^Faculté de Pharmacie, Institut sur le Vieillissement et la Participation Sociale des Aînés and Institut sur la Nutrition et les Aliments Fonctionnels (INAF), Université Laval, Montreal, ON, Canada; ^12^Montreal Heart Institute Research Center, and Département de Nutrition, Université de Montréal, Montreal, QC, Canada

**Keywords:** cardiovascular risk factors, cognition, older adults, cognitively healthy, HDL

## Abstract

**Introduction:**

Cardiovascular disease risk factors (CVRFs) contribute to the development of cognitive impairment and dementia.

**Methods:**

This study examined the associations between circulating CVRF biomarkers and cognition in 386 cognitively healthy older adults (mean age = 78 ± 4 years, 53% females) selected from the Quebec Longitudinal Study on Nutrition and Successful Aging (NuAge). Memory, executive function, and processing speed were assessed at baseline and 2-year follow-up. CVRF biomarkers included total cholesterol, high-density lipoprotein cholesterol (HDL-C), low-density lipoprotein-cholesterol (LDL-C), triglycerides, glucose, insulin, high sensitivity C-reactive protein (hs-CRP), homocysteine, protein carbonyls, and cortisol. Linear mixed models were used to determine associations between individual CVRF biomarkers and cognition at both time points.

**Results:**

HDL-C was most consistently associated with cognition with higher values related to better performance across several domains. Overall, stronger and more consistent relationships between CVRF biomarkers and cognition were observed in females relative to males.

**Discussion:**

Findings suggest that increases in the majority of circulating CVRFs are not associated with worse cognition in cognitively healthy older adults.

## Highlights

- HDL-C was shown to be the most robust positive associations with cognition.
- Associations between CVRFs and cognition were observed primarily in females.
- Most CVRFs were not negatively associated with cognition in healthy older adults.

## 1. Introduction

As worldwide age expectancy increases, there is a rising prevalence of Alzheimer’s disease (AD) and related dementias; conditions characterized by cognitive impairment, resulting in a loss of independence and decreased quality of life for older adults. Cardiovascular risk factors (CVRFs) contribute to the development of cognitive impairment and dementia (Livingston et al., 2020). Circulating CVRF biomarkers such as blood glucose, cholesterol, and triglyceride levels are routinely measured in clinical practice to assess overall cardiovascular health, and there is much speculation that these markers can also serve as risk factors for cognitive impairment (Lai et al., 2020). Impairments in cognition with a vascular origin are predominantly deficits in executive function and processing speed; however, cognitive performance across individuals is quite variable (Gorelick et al., 2011). A better understanding of the links between certain CVRF biomarkers and specific cognitive functions may help to inform dementia risk reduction strategies.

Adhering to a healthy diet is considered a prudent strategy for mitigating cognitive decline beyond what is associated with normal aging. Consumption of high-quality diets centered on plant-source foods, whole grains, and fish, such as the Mediterranean diet, associate with lower risk of mild cognitive impairment and dementia (Singh et al., 2014). Research stemming from the Quebec Longitudinal Study on Nutrition and Successful Aging (NuAge), demonstrates greater preservation of cognitive function in older adults consuming higher quality diets (Parrott et al., 2021). Evidence points to the neuroprotective effects of high-quality diets on lowering CVRFs; with inflammation and oxidative burden being primary mechanisms (Parrott and Greenwood, 2007; Frisardi et al., 2010). For instance, higher quality diets reduce chronic diseases associated with dementia risk such as the metabolic syndrome, which represents a collective of risk factors {obesity, high blood pressure, dyslipidemia [elevated triglycerides and lower high-density lipoprotein-cholesterol (HDL-C)], and insulin resistance; Mayo Clinic, 2022}. Identifying which biomarkers are predictors of cognitive function may also help to understand the mediators of diet-associated cognitive changes and help to evaluate the impact of dietary interventions (Fiocco et al., 2019).

Much of the research investigating associations between circulating CVRF biomarkers and cognition has been conducted in individuals with established cardiovascular disease and/or various forms of cognitive impairment. Less is known, however, about the cognitive effects of these markers in cognitively healthy older adults. The objective of this study was 2-fold: (1) to explore the associations between circulating CVRF biomarkers and cognitive performance at baseline and 2-year follow-up in a sample of cognitively healthy older adults from the NuAge Study; and (2) to explore sex differences in CVRF-cognitive associations.

## 2. Materials and methods

### 2.1. Study design/participants

NuAge is a prospective cohort study of 1,793 cognitively healthy older adults aged 67–84 years at baseline. Participants were recruited from a random sample of the Quebec Medicare database, stratified for age and sex, and 1,753 (98%) consented to integrate their data into the NuAge Database and Biobank for future studies. Participants were evaluated four times over a 5-year period at the Institut universitaire de gériatrie de Montréal or the Institut universitaire de gériatrie de Sherbrooke in 2003–2005 (NuAge T1), 2005–2006 (NuAge T2), 2006–2007 (NuAge T3), and 2007–2008 (NuAge T4).

In 2006–2008, a subsample of 461 participants were recruited to the Nutrition and Cognition (NutCog) sub-study, which entailed completion of a neuropsychological test battery at baseline (NutCog T1) and 2-year follow-up (NutCog T2; Duchaine et al., 2022). At time of recruitment, NutCog participants were 70–86 years with a Modified Mini-Mental State (3MS; Teng and Chui, 1987) score of >85/100. NutCog T1 cognitive assessments were conducted in 2006–2008, 2–3 months preceding or proceeding the closest annual NuAge evaluation (i.e., either NuAge T3 or T4); and NutCog T2 assessments were conducted at 2-year follow up from NutCog T1 in 2008–2010.

Of the 461 NutCog participants, 36 were excluded due to the presence of self-reported conditions that may impair cognition (i.e., dementia, Parkinson disease, epilepsy, and history of stroke), and 39 were removed due to missing data on circulating CVRF biomarker concentrations (Presse et al., 2013; Duchaine et al., 2022). Accordingly, the analytic sample comprised 386 participants at NutCog T1, of which 321 participants were evaluated at NutCog T2 (16.8% attrition). The NuAge study and the embedded NutCog study protocols were approved by the Research Ethics Boards of the Institut universitaire de gériatrie de Montréal and the Institut universitaire de gériatrie de Sherbrooke (Quebec, Canada). The NuAge Database and Biobank have been approved by the Research Ethic Board of the CIUSSS de l’Estrie–CHUS (Quebec, Canada). All participants provided informed consent to NuAge and NutCog studies.

### 2.2. CVRF biomarkers

Cardiovascular risk factor biomarkers included lipids [serum total cholesterol (TC), high-density lipoprotein cholesterol (HDL-C), low-density lipoprotein cholesterol (LDL-C), and triglycerides (TG)], which serve as an indicator of metabolic status and are associated with late-life cognitive impairment and Alzheimer’s disease (Reitz and Mayeux, 2014); blood glucose, serum insulin, and insulin resistance calculated using the homeostatic assessment of insulin resistance (HOMA-IR), which are indicators associated with dementia risk via various mechanisms including compromised cerebrovascular health and brain insulin metabolism signaling (Parrott and Greenwood, 2007; Craft et al., 2012; Meusel et al., 2014); plasma homocysteine which is negatively associated with cognitive function through various vascular and oxidative mechanisms (Schulz, 2007); serum high sensitivity C-reactive protein (hs-CRP), an indicator of inflammation that has emerged as an important factor in atherosclerosis (Sartori et al., 2012) and as a risk factor for cognitive decline (Laurin et al., 2009); plasma protein carbonyls, an indicator of oxidative and protein damage (Dalle-Donne et al., 2003); and salivary cortisol (the difference between morning and evening levels), a stress hormone that contributes to cardiovascular risk (Whitworth et al., 2005) and has been associated with cognitive dysfunction (Maggio et al., 2013).

### 2.3. Biomarker sampling

Blood sampling, processing, and storing in the NuAge Study followed procedures which have been described elsewhere (Gaudreau et al., 2007). At annual interviews, a 50-mL blood sample was obtained between 7:30 and 8:30 AM after an overnight fast. Specifically, participants were instructed not to eat, drink (except reasonable amount of water), smoke, or chew gum after 7:00 PM the evening before the blood sampling. Specimens were extracted from fresh blood collected in tubes containing a coagulation activator (serum) and EDTA as an anticoagulant (plasma). Samples were then aliquoted in 1.5-mL opalescent-colored Eppendorf polypropylene tubes, stored at −20 degrees Celcius (C) in opaque cardboard for few hours, and then at −80°C for long-term storage. Overall, light exposure was limited to periods of sample processing, for a total light exposure time of about 100 min.

Serum TC, HDL-C, and TG concentrations were analyzed by the Center hospitalier de l’Université de Montreal clinical biochemistry laboratory using COBAS C-311 platform and TRIGL, CHOL2 and HDLC3 kits (Roche Diagnostic Corp. Indianapolis, IN, United States; Presse et al., 2013). LDL-C was obtained using the Friedewald equation: LDL-C (mmol/L) = TC (mmol/L) − HDL-C (mmol/L) − TG (mmol/L)/2.2 (Friedewald et al., 1972; Tremblay et al., 2004). Glucose concentrations were determined using a standardized automated method (glucose oxidase; Gaudreau et al., 2007). Insulin was assessed using a human insulin radioimmunoassay (RIA) kit (detection limit: 12 pmol/L; intra-assay coefficients of variation: 1.1–8.3%; Linco Research Inc., St. Charles, MO, United States). Insulin resistance was estimated based on the HOMA-IR using the following formula: fasting serum insulin (mU/mL) × fasting serum glucose (mmol/L)/22.5 (Gaudreau et al., 2007; Matta et al., 2016). Plasma total homocysteine and hs-CRP were analyzed at the Center hospitalier de l’Université de Montreal clinical biochemistry laboratory. Total homocysteine levels were quantified by high pressure liquid chromatography with fluorescence detection. The intra- and inter-assay coefficients of variation were 3.6 and 4.1%, respectively. hs-CRP levels were quantified using the highly sensitive CardioPhase competitive immunoassay (Siemens Healthcare Limited, Oakville, ON, CA) coupled with nephelometric detection. The intra- and inter-assay coefficients of variation were 4.4 and 5.7%, respectively. Fasting morning and evening salivary cortisol concentrations were measured using the salivary cortisol EIA kit from Salimetrics (Carlsbad, CA). The intra- and inter-assay coefficients of variation were 8.0 and 1.6%, respectively. Protein carbonyl (PCO) were quantified in our laboratories by spectrophotometry after derivatization with 2,4-dinitrophenylhydrazine (Sigma-Aldrich, Saint-Louis, MO, United States). The intra- and inter-assay coefficients of variation were 7.5 and 7.2%, respectively.

### 2.4. Cognitive assessment

To measure performance in verbal and non-verbal episodic memory, executive functioning, and processing speed, 13 scores from six validated tests were used. Individual cognitive test scores were standardized (Z-transformed) to the sample mean and summed to calculate a total *Z*-score for each cognitive domain (Duchaine et al., 2022). Cognitive scores at follow-up were standardized to the mean at baseline in order to reflect cognitive change over the 2-year period. Verbal episodic memory included total scores from the three-immediate free recall trials and the 20-min delayed free recall trial of the RL/RI-16 Free and Cued Recall Task (RL/RI-16 FCRT; Van der Linden et al., 2004). Non-verbal episodic memory included scores from the 3-min and 20-min recalls of the Rey Complex-Figure task (Rey, 1959). Executive functioning was assessed using the scores from the Rey Complex-Figure copying task (Rey, 1959), the difference in reaction time (RT) between the first and third condition of the Stroop Test (Spreen and Strauss, 1991), the total number of letters recalled in the interference conditions (10-, 20-, and 30-s delays) of an Adapted Brown-Peterson procedure (Spreen and Strauss, 1998), and the total number of completed targets in the Digit Symbol-Coding subtest of the Wechsler Adult Intelligence Scale-III (Wechsler, 1997). Processing speed included RT on the congruent naming condition of the Stroop Test (i.e., name the printed color of the dot (plate 1); Spreen and Strauss, 1991) and the mean RT in the Choice-Reaction Time task (Cohen et al., 1993). Due to departures from symmetry, all raw RTs included in processing speed were log-transformed and multiplied by −1 so that larger values always indicated better performances. The processing speed domain score is computed by summing the standardized test scores following transformation.

### 2.5. Covariates

Our analyses accounted for potential socio-demographic, lifestyle, and comorbidity confounders known to impact brain health. Data for age, body mass index (BMI, kg/m^2^), diet quality, physical activity, and depressive symptoms were obtained at the time of NutCog T1. Healthy diet intake was assessed using the Canadian Healthy Eating Index (C-HEI, Shatenstein et al., 2005); physical activity was evaluated with the Physical Activity Scale for the Elderly (PASE; Washburn et al., 1999); and depressive symptoms were evaluated with the Geriatric Depression Scale (GDS; Yesavage et al., 1982). Higher scores on each of the questionnaires indicated greater endorsement of the respective factor. Data for sex (male/female), number of years of education, smoking status (never or current/former smokers), and presence of hypertension (yes/no; self-reported physician diagnosis) were collected from baseline interviews of the NuAge study.

### 2.6. Statistical analyses

Differences in baseline characteristics (covariates) between males and females were analyzed using independent *t*-test for continuous variables and chi squared test for categorical variables. To check for biases from nonresponse and losses to follow-up, these same tests were conducted to compare baseline CVRF levels and cognitive performance between individuals who completed the study and individuals who were lost to follow-up.

Linear models were conducted to estimate the slopes of relationships between CVRF biomarkers (*n* = 11) and cognition, both measured at two time-points. NutCog T1 cognitive assessment was matched to NuAge blood/saliva sampling 2 years prior (either NuAge T1 or T2) and NutCog T2 cognitive assessment was matched to the NuAge blood/saliva sampling at the time of NutCog T1 (either NuAge T3 or T4; Appendix Table A1). The median time delay between matched NuAge and NutCog assessments at both time-points was 2.2 years.

For each domain, 11 repeated measures regression models (one for each CVRF) were fitted to the data with an unstructured error covariance matrix to account for intra-individual correlation, and were then adjusted for potential covariates. The unadjusted model included time, biomarker, and their interaction. The adjusted model further included age, sex, education, BMI, smoking status, diet quality, physical activity, presence of hypertension, GDS scores, and the interaction term between sex and biomarker. Missing values on covariates resulted in a slightly decreased sample size in the fully adjusted model.

Overall relationships between biomarkers and cognitive domains were tested using Type 3 tests. Given the attrition rate at follow-up, we did not model cognitive decline directly, but investigated how the associations between CVRF biomarkers and cognition changed over time (CVRF x time interaction) as the linear mixed models allow for incomplete follow-up. As the focus was to compare estimated slopes and confidence intervals between variables, all associations with an overall (type 3 test) value of *p* < 0.10 were highlighted.

When multiple CVRFs were associated with a particular cognitive domain, multivariate models were constructed to determine the combined effects of those biomarkers on cognition. When overall associations or sex interactions were present, sex stratified regression analyses were conducted for those models. Furthermore, sensitivity analyses were conducted to determine how certain unexpected relationships differed when individuals with biomarker values above clinical cut-offs (glucose ≥7.0 mmol/L, hs-CRP ≥ 10.0 mg/L) were removed from the final models.

## 3. Results

### 3.1. Participant characteristics

Baseline characteristics of the 386 participants, overall and stratified by sex, are shown in Table 1. Overall, the mean age (SD) was 77.6 (3.8) years, approximately 53% of the sample was females, and the mean number of years of education was 13.0 (4.5). Compared to males, females had lower levels of education, physical activity, and were less likely to be a current smoker. Females also had greater depressive symptoms relative to males, better diet quality, and a greater percentage of females was classified as having hypertension. No sex differences in age and BMI were observed. Table 2 shows mean CVRF biomarker values, mean cognitive test scores, and cognitive performance (summed *Z*-scores) by domain at baseline and follow-up. A total of 71 participants (45% females) did not complete the cognitive assessment at follow-up. Individuals who were lost to follow-up had lower cognitive performance across all domains at baseline compared to individuals who completed the study (Appendix Table A1).

**Table 1:** Baseline characteristics of the study sample.

	Whole sample	Males	Females	*p* values
	*n* = 386	*n* = 183	*n* = 203	
Age (years)	77.6 ± 3.8	77.4 ± 3.7	77.9 ± 3.9	0.228
Education (years)	13.0 ± 4.5	14.0 ± 4.6	12.1 ± 4.2	<0.001
Body mass index, (kg/m^2^)	27.8 ± 4.7	28.1 ± 4.2	27.6 ± 5.1	0.341
*Missing*	1 (0.3)	0 (−)	1 (0.5)	
Diet quality (C-HEI)	72.1 (11.7)	69.4 (11.9)	74.5 (11.0)	<0.001
*Missing*	3 (0.8)	3 (1.6)	0 (−)	
Physical activity (PASE score)	95.3 ± 47.5	112.4 ± 52.1	79.7 ± 36.5	0.003
*Missing*	18 (4.7)	7 (3.8)	11 (5.4)	
Smoking status—current or former	169 (43.8)	119 (65.0)	50 (24.6)	<0.001
Hypertension (yes)	204 (53.1)	91 (50)	113 (55.9)	0.244
*Missing*	2 (0.5)	1 (0.5)	1 (0.5)	
Number of depressive symptoms (GDS)	4.2 ± 3.7	3.6 ± 3.4	4.7 ± 3.8	0.003
*Missing*	6 (1.6)	1 (0.5)	5 (2.5)	

Data presented as means ± standard deviation or *n* (%). C-HEI, Canadian healthy eating index; PASE, Physical activity scale for the elderly; GDS, Geriatric depression scale.

**Table 2:** CVRF biomarker levels and cognitive performance at baseline and follow-up.

CVRF biomarker levels	NuAge	NuAge
	T1 or T2	T3 or T4
Total cholesterol (mmol/L)	5.09 ± 0.93	5.31 ± 1.07
HDL-C (mmol/L)	1.34 ± 0.37	1.39 ± 0.39
LDL-C (mmol/L)	3.04 ± 0.81	3.16 ± 0.92
Triglycerides (mmol/L)	1.58 ± 0.74	1.68 ± 0.83
Fasting glucose (mmol/L)	5.57 ± 1.16	5.38 ± 1.06
Insulin (pmol/L)	19.63 ± 15.64	17.98 ± 16.50
HOMA-IR	4.97 ± 4.01	4.48 ± 4.28
Homocysteine (mcmol/L)	12.88 ± 4.14	13.41 ± 4.61
hs-CRP (mg/L)	2.73 ± 3.41	3.29 ± 9.87
Cortisol (mcg/dL)	3.19 ± 6.71	4.07 ± 21.17
Protein carbonyls (nmol/mg)	2.04 ± 1.33	1.93 ± 1.23
Cognitive performance	NutCog	NutCog
Verbal episodic memory (words recalled)	T1	T2
First free recall	7.31 ± 2.39	7.53 ± 2.19
Second free recall	9.21 ± 2.80	9.17 ± 2.55
Third free recall	10.61 ± 2.96	10.37 ± 2.71
20 min delayed recall	11.44 ± 3.00	11.29 ± 2.88
Total *Z*-score	0 ± 3.50	−0.06 ± 3.25
Non-verbal episodic memory		
Rey Complex figure—3 min delay (out of 36)	17.97 ± 5.24	14.14 ± 4.75
Rey Complex figure—20 min delay (out of 36)	17.55 ± 5.01	14.24 ± 4.76
Total *Z*-score	0 ± 1.95	−1.39 ± 1.80
Executive function		
Rey complex figure—copying (out of 36)	30.30 ± 2.98	26.83 ± 3.92
Stroop test—difference of plate 1 and 3 (time (s))	22.71 ± 12.01	22.04 ± 12.02
Brown-Peterson test (total letters recalled)	19.87 ± 5.30	17.82 ± 5.66
Digit-symbol coding (number completed)	47.84 ± 12.13	46.12 ± 11.80
Total *Z*-score	0 ± 2.63	−1.62 ± 2.83
Processing speed		
Stroop test condition 1 [reading time (s)]	14.98 ± 3.58	14.51 ± 3.80
Choice reaction time [initiation time (ms)]	472.44 ± 88.30	468.51 ± 90.67
Total *Z*-score	0 ± 1.54	0.19 ± 1.54

Means ± SD. Individual cognitive test performances are represented by raw scores. Performances in cognitive domains are represented by *Z*-scores. *Z* scores at follow-up are standardized to the mean and SD at baseline. HDL-C, High density lipoprotein–cholesterol; LDL-C, Low density lipoprotein-cholesterol; HOMA-IR, the Homeostatic model assessment for insulin resistance; and hs-CRP, High sensitivity C-reactive protein.

### 3.2. Cognition

Table 3 displays the whole sample slopes and confidence intervals representing the associations between individual CVRF biomarkers and cognitive domain Z-scores in adjusted models at both time-points. The slopes and confidence intervals of unadjusted models are presented in the Appendix Table A2. Table 4 displays the slopes and confidence intervals in males and females separately for biomarkers that showed an overall association with cognitive domains, or when sex interactions were present in univariate analyses. Table 5 displays the whole sample slopes and confidence intervals representing the associations between CVRF’s and cognitive domains in multivariate models. Results pertaining to the adjusted models for each cognitive domain are described below.

**Table 3:** Associations between CVRF biomarkers and cognition at baseline and follow-up among the whole sample.

	*β* (95% CI)	*β* (95% CI)
	Verbal episodic memory	Non-verbal episodic memory
Total cholesterol^1^	0.24 (−0.13; 0.61)	0.01 (−0.21; 0.23)
Total cholesterol^2^	0.09 (−0.25; 0.44)	−0.03 (−0.23; 0.17)
HDL-C^1^	**1.67 (0.71; 2.64)**	**0.76 (0.18; 1.34)**
HDL-C^2^	0.31 (−0.70; 1.32)^*^^	−0.16 (−0.77; 0.44)^^†^
LDL-C^1^	0.11 (−0.31; 0.52)	−0.07 (−0.32; 0.18)
LDL-C^2^	0.09 (−0.30; 0.48)	0.03 (−0.20; 0.27)
Triglycerides^1^	−0.34 (−0.80; 0.12)	−0.20 (−0.48; 0.07)
Triglycerides^2^	−0.10 (−0.36; 0.56)^†^	−0.14 (−0.41; 0.14)^*^
Glucose^1^	0.21 (−0.10; 0.52)	−0.12 (−0.31; 0.07)
Glucose^2^	0.31 (−0.07; 0.69)^*†^	−0.03 (−0.26; 0.20)
Insulin^1^	−0.00 (−0.03; 0.03)	−0.00 (−0.02; 0.02)
Insulin^2^	0.00 (−0.03; 0.03)	−0.01 (−0.02; 0.01)
HOMA-IR^1^	0.02 (−0.09; 0.12)	−0.00 (−0.07; 0.06)
HOMA-IR^2^	0.03 (−0.06; 0.12)	−0.02 (−0.07; 0.04)
Homocysteine^1^	−0.02 (−0.11; 0.06)	−0.01 (−0.06; 0.04)
Homocysteine^2^	−0.02 (−0.06; 0.11)	0.02 (−0.03; 0.07)
hs-CRP^1^	0.03 (−0.13; 0.08)	0.01 (−0.05; 0.08)
hs-CRP^2^	0.04 (−0.03; 0.11)	0.00 (−0.04; 0.04)
Cortisol^1^	0.01 (−0.04; 0.07)	0.01 (−0.02; 0.04)
Cortisol^2^	0.02 (−0.02; 0.06)	0.02 (−0.01; 0.04)
Protein carbonyls^1^	**0.24 (0.00; 0.49)**	−0.04 (−0.19; 0.10)
Protein carbonyls^2^	−0.19 (−0.48; 0.10)^**^**†^	−0.04 (−0.21; 0.14)
	Executive function	Processing speed
Total cholesterol^1^	0.16 (−0.13; 0.44)	0.08 (−0.09; 0.27)
Total cholesterol^2^	0.17 (−0.09; 0.43)	−0.01 (−0.17; 0.16)
HDL-C^1^	0.69 (−0.06; 1.43)	−0.20 (−0.67; 0.26)
HDL-C^2^	0.77 (−0.00; 1.54)^*^	−0.11 (−0.59; 0.37)
LDL-C^1^	0.04 (−0.27; 0.36)	0.13 (−0.07; 0.33)
LDL-C^2^	0.16 (−0.13; 0.46)	0.02 (−0.16; 0.21)
Triglycerides^1^	−0.10 (−0.46; 0.25)	0.05 (−0.17; 0.27)
Triglycerides^2^	−0.28 (−0.63; 0.07)	−0.08 (−0.30; 0.14)
Glucose^1^	−0.04 (−0.28; 0.20)	−0.05 (−0.19; 0.10)
Glucose^2^	0.03 (−0.26; 0.32)	−0.01 (−0.19; 0.16)
Insulin^1^	−0.00 (−0.03; 0.02)	0.00 (−0.01; 0.01)
Insulin^2^	0.00 (−0.02; 0.02)	0.00 (−0.01; 0.02)
HOMA-IR^1^	−0.01 (−0.08; 0.07)	−0.00 (−0.05; 0.04)
HOMA-IR^2^	0.01 (−0.06; 0.08)	0.01 (−0.03; 0.05)
Homocysteine^1^	−0.05 (−0.12; 0.14)	−0.01 (−0.05; 0.03)
Homocysteine^2^	−0.04 (−0.10; 0.03)^*^	−0.02 (−0.06; 0.02)
hs-CRP^1^	0.07 (−0.02; 0.15)	0.03 (−0.03; 0.08)
hs-CRP^2^	**0.07 (0.01; 0.12)** ^*†^	**0.04 (0.01; 0.07)** ^*†^
Cortisol^1^	0.02 (−0.03; 0.06)	−0.00 (−0.03; 0.02)
Cortisol^2^	0.01 (−0.03; 0.04)	0.01 (−0.01; 0.03)
Protein carbonyls^1^	0.13 (−0.06; 0.32)	0.08 (−0.11; 0.12)
Protein carbonyls^2^	0.14 (−0.08; 0.35)^*^	−0.02 (−0.16; 0.11)^†^

CVRF, Cardiovascular risk factor; HDL-C, High density lipoprotein cholesterol; ^1^Baseline; ^2^Follow-up; LDL-C, Low density lipoprotein cholesterol; HOMA-IR, Homeostatic model assessment for insulin resistance; and hs-CRP, High sensitivity C-reactive protein. ^*^Overall association (type 3 test *p* < 0.10); ^†^Sex interaction (*p* < 0.10); ^Time interaction (*p* < 0.10). Bolded data denote associations with confidence intervals that do not include zero. Models are adjusted for time; CVRF by time interaction; age; sex; CVRF by sex interaction; education; BMI; diet quality (C-HEI); physical activity (PASE); smoking status; hypertension; and depressive symptoms (GDS).

**Table 4:** Sex stratified analyses of univariate models showing associations between CVRF biomarkers and cognitive domains at baseline and follow-up.

	Males	Females
	*β* (95% CI)	*β* (95% CI)
Verbal episodic memory		
HDL-C^1^	1.52 (−0.21; 3.25)	**1.75 (0.57; 2.94)**
HDL-C^2^	0.10 (−1.73; 1.93)	0.29 (−0.93; 1.51)^*^^
Triglycerides^1^	0.16 (−0.53; 0.87)	**−0.68 (−1.30; −0.07)**
Triglycerides^2^	0.49 (−0.33; 1.31)	−0.21 (−0.74; 0.32)^*^
Glucose^1^	0.02 (−0.38; 0.41)	0.39 (−0.10; 0.87)
Glucose^2^	−0.07 (−0.62; 0.49)	**0.67 (0.13; 1.20)** ^*^
Protein carbonyls^1^	**0.45 (0.09; 0.82)**	−0.01 (−0.34; 0.32)
Protein carbonyls^2^	−0.11 (−0.55; 0.32)^^^	−0.27 (−0.65; 0.12)
Non-verbal episodic memory		
HDL-C^1^	−0.16 (−1.22; 0.89)	**1.49 (0.80; 2.19)**
HDL-C^2^	−0.20 (−1.32; 0.91)	0.15 (−0.57; 0.87)^*^^
Triglycerides^1^	−0.01 (−0.44; 0.42)	**−0.38 (−0.75; −0.02)**
Triglycerides^2^	−0.29 (−0.78; 0.21)	−0.11 (−0.43; 0.20)^*^
Executive function		
HDL-C^1^	−0.04 (−1.29; 1.21)	**1.28 (0.32; 2.24)**
HDL-C^2^	0.24 (−1.08; 1.55)	**1.37 (0.38; 2.36)** ^*^
Homocysteine^1^	−0.06 (−0.14; 0.02)	−0.05 (−0.16; 0.07)
Homocysteine^2^	−0.04 (−0.11; 0.04)^*^	−0.03 (−0.15; 0.09)
hs-CRP^1^	0.01 (−0.10; 0.11)	0.11 (−0.02; 0.24)
hs-CRP^2^	0.00 (−0.03; 0.04)	**0.12 (0.00; 0.24)** ^*^
Protein carbonyls^1^	0.11 (−0.16; 0.37)	0.15 (−0.11; 0.41)
Protein carbonyls^2^	0.14 (−0.17; 0.46)	0.14 (−0.17; 0.45)
Processing speed		
hs-CRP^1^	−0.02 (−0.08; 0.05)	0.06 (−0.02; 0.14)
hs-CRP^2^	0.01 (−0.01; 0.04)	0.05 (−0.02; 0.12)^*^
Protein carbonyls^1^	0.03 (−0.13; 0.20)	−0.02 (−0.19; 0.13)
Protein carbonyls^2^	0.17 (−0.03; 0.37)	**−0.21 (−0.39; −0.02)** ^*^

CVRF, Cardiovascular risk factor; HDL-C, High density lipoprotein-cholesterol; hs-CRP, High sensitivity C-reactive protein. ^*^Overall association (type 3 test *p* < 0.10); ^^^Time interaction (*p* < 0.10). Bolded data denote associations with confidence intervals that do not include zero. Models are adjusted for time; CVRF by time interaction; age; education; BMI; diet quality (C-HEI); physical activity (PASE); smoking status; hypertension; and depressive symptoms (GDS).

**Table 5:** Multivariate analysis showing specific associations between CVRF biomarkers and cognitive domains at baseline and follow-up.

	Whole sample^a^	Males^b^	Females^b^
	*β* (95% CI)	*β* (95% CI)	*β* (95% CI)
Verbal episodic memory			
HDL-C^1^	**1.76 (0.79; 2.73)**	1.58 (−0.17; 3.33)	**1.99 (0.83; 3.15)**
HDL-C^2^	0.29 (−0.73; 1.31)^*^^	0.03 (−1.84; 1.90)	0.19 (−1.02; 1.39)^*^^
Glucose^1^	0.28 (−0.03; 0.58)	0.04 (−0.36; 0.45)	0.47 (−0.01; 0.95)
Glucose^2^	0.28 (−0.10; 0.66)^*†^	−0.11 (−0.67; 0.46)	**0.64 (0.12; 1.16)** ^*^
Executive function			
HDL-C^1^	0.78 (−0.04; 1.59)	0.03 (−1.32; 1.38)	**1.44 (0.37; 2.51)**
HDL-C^2^	0.71 (−0.09; 1.50)^*^	0.20 (−1.15; 1.55)	**1.35 (0.33; 2.36)** ^*^
hs-CRP^1^	0.07 (−0.02; 0.15)	0.01 (−0.10; 0.12)	0.09 (−0.04; 0.22)
hsCRP^2^	**0.07 (0.02; 0.12)** ^*†^	0.00 (−0.03; 0.04)	0.12 (−0.01; 0.24)^*^

CVRF, Cardiovascular risk factor; HDL-C, High density lipoprotein-cholesterol; hs-CRP, High sensitivity C-reactive protein. ^*^Overall association (type 3 test *p* < 0.10); ^†^Sex interaction (*p* < 0.10); ^^^Time interaction (*p* < 0.10). Bolded data denote associations with confidence intervals that do not include zero. ^a^Models are adjusted for time; CVRF by time interaction; age; sex; CVRF by sex interaction; education; BMI; diet quality (C-HEI); physical activity (PASE); smoking status; hypertension; and depressive symptoms (GDS). ^b^Models are adjusted for time; CVRF by time interaction; age; education; BMI; diet quality (C-HEI); physical activity (PASE); smoking status; hypertension; and depressive symptoms (GDS).

#### 3.2.1. Verbal episodic memory

Verbal episodic memory was positively associated with HDL-C (type 3 *p* = 0.010) and glucose (type 3 *p* = 0.041) overall (Table 3). An HDL by time interaction (*p* = 0.036) reflected a stronger and more consistent association between HDL-C and verbal episodic memory at baseline, compared to follow-up (Table 3). Sex differences were indicated for relationships with glucose, protein carbonyls, and triglycerides (sex interaction *p* values between 0.055 and 0.083). Sex stratified results depicting the relationships of HDL-C, glucose, protein carbonyls, and triglycerides with verbal episodic memory are displayed in Table 4.

In a fully adjusted multivariate model including HDL-C and glucose as predictors, both HDL-C (type 3 *p* = 0.008) and glucose (type 3 *p* = 0.030) were positively associated with verbal episodic memory overall. HDL-C by time (*p* = 0.026) and glucose by sex interactions (*p* = 0.054) also remained. There was a strong and consistent relationship between HDL-C and verbal memory at baseline, but not follow-up (Table 5). When stratified by sex, positive relationships between fasting glucose and verbal memory at both time-points were observed in females (type 3 *p* = 0.003), but not males (type 3 *p* = 0.857; Table 5). Sensitivity analyses removing individuals with glucose levels greater than 7.0 mmol/L (*n* = 17) eliminated sex differences in the relationship between glucose and verbal memory (Appendix Table A3).

#### 3.2.2. Non-verbal episodic memory

Non-verbal episodic memory was negatively associated with triglycerides (type 3 *p* = 0.092) overall, however the associations at baseline and follow-up were relatively weak and inconsistent (Table 3). Negative associations were observed in both sexes, but were stronger and more consistent in females (type 3 *p* = 0.048; Table 4). Though no overall association with HDL-C was observed, HDL-C by time (*p* = 0.017) and HDL-C by sex (*p* = 0.072) interactions were present. HDL-C was positively associated with non-verbal episodic memory at baseline, but not follow-up (Table 3). When stratified by sex, this relationship was observed only in females (Table 4).

#### 3.2.3. Executive function

Executive function was positively associated with HDL-C (type 3 *p* = 0.014), hs-CRP (type 3 *p* = 0.011), and protein carbonyls (type 3 *p* = 0.071) overall (Table 3). A hs-CRP by sex interaction was identified (*p* = 0.012). Executive function was negatively associated with homocysteine (overall *p* = 0.078). Sex stratified univariate results depicting the relationships of HDL-C, hs-CRP, protein carbonyls, and homocysteine with executive function are displayed in Table 4.

HDL-C, hs-CRP, homocysteine, and protein carbonyls were brought forward as predictors in a fully adjusted multivariate model, however protein carbonyls and homocysteine did not contribute to the model (Appendix Table A4) and were removed in a stepwise fashion. Executive function remained positively associated with both HDL-C (type 3 *p* = 0.018) and hs-CRP (type 3 *p* = 0.010) overall (Table 5). A hs-CRP by sex interaction (*p* = 0.013) also remained. When stratified by sex, significant associations between executive function and HDL-C at baseline and follow-up were observed in females (overall *p* = 0.001), but not males (overall *p* = 0.825). Stronger and more consistent associations between executive function and hs-CRP were observed in females (overall *p* = 0.017), compared to males (overall *p* = 0.817). Sensitivity analyses removing individuals with hs-CRP levels greater than 10.0 mmol/L (*n* = 4) eliminated sex differences in the relationship between hs-CRP and executive function (Appendix Table A3).

#### 3.2.4. Processing speed

Processing speed was positively associated with CRP (overall *p* = 0.036). Hs-CRP by sex (*p* = 0.060) and protein carbonyl by sex (*p* = 0.024) interactions were observed. When stratified by sex, more consistent associations between processing speed and hs-CRP were observed in females (overall *p* = 0.062), compared to males (overall *p* = 0.932; Table 4). Negative associations between protein carbonyls and processing speed were observed in females (overall *p* = 0.059) but not males (overall *p* = 0.131; Table 4). Sensitivity analyses removing individuals with hs-CRP levels greater than 10.0 mmol/L (*n* = 4) eliminated sex differences in the relationship between hs-CRP and processing speed (Appendix Table A3).

## 4. Discussion

This study investigated associations between CVRF biomarkers and cognition in cognitively healthy older adults. HDL-C was most consistently associated with cognition across the whole sample, and more associations between biomarkers and cognition were observed in females, compared to males. Sensitivity analyses removing individuals with high glucose and CRP values attenuated sex differences. The current results suggest that the majority of circulating CVRFs are not associated with cognition in cognitively healthy older adults.

### 4.1. HDL-C associations

The associations between HDL-C and cognition observed in our study is consistent with the literature suggesting that higher levels of HDL-C support cognitive health and may minimize risk of dementia (Barzilai et al., 2006; Reitz et al., 2010; Svensson et al., 2019). Several cross-sectional studies have observed positive associations between HDL-C levels, memory, and executive function in cognitively unimpaired older adults (Song et al., 2012; Crichton et al., 2014; Ihle et al., 2017; Sun et al., 2019). In addition to playing a major role in the cholesterol transport pathway, HDL-Cs functions include anti-oxidant, anti-inflammatory, anti-thrombotic roles, and endothelial- and immune-modulatory function (Bonacina et al., 2021), all of which support brain health. There is strong evidence that individuals who eat healthier and engage in regular exercise have higher HDL-C levels (Sanllorente et al., 2021; Franczyk et al., 2023). High-quality protein and n-3 poly-unsaturated fatty acids derived from vegetable, nut, and fish intake help to increase HDL-C levels, while exercise enhances HDL-C levels and exerts a beneficial impact on HDL composition and functionality (Alhassan et al., 2017; Franczyk et al., 2023). Engaging in lifestyle behaviors that promote increases in HDL-C may be a worthwhile strategy for older adults to preserve memory function with aging.

### 4.2. Glucose and CRP associations

The positive relationship between glucose and verbal episodic memory observed in our study is not consistent with the extant literature. A quadratic trend test was conducted to further explore this unexpected linear relationship; however, no significant non-linear trends were observed (results not shown). Some studies however, have observed positive associations between fasting glucose and memory (Hall et al., 1989; Riby et al., 2004; Sims Wright et al., 2015). Glucose ingestion has been found to enhance memory performance in younger and older adults (Hall et al., 1989; Riby et al., 2004). In a study of healthy non-diabetic younger adult females, blood glucose was associated with better cognitive performance on highly challenging tasks, but not with less challenging tasks (Donohoe and Benton, 2000). Similarly, among non-diabetic adults 70 years of age and older, higher fasting glucose was associated with better verbal memory (Sims Wright et al., 2015). Although mean fasting glucose was slightly lower in this sample compared with the current study (5.23 ± 0.49 vs. 5.57 ± 1.16 mmol/L), mean values in both studies were below the diabetic range. Removing individuals with glucose levels higher than 7 mmol/L from our analysis revealed a more robust glucose-cognition relationship. As the brain relies primarily on glucose for positive energy (Oz et al., 2007), it may be possible that in healthy older adults, increased fasting glucose levels contribute to better memory when values remain below clinical risk levels.

The association between hs-CRP and cognition in older adults is unclear. Some studies suggest that higher hs-CRP levels are associated with cognitive impairments and increased risk of cognitive decline in older adults (Laurin et al., 2009; Noble et al., 2010; Weinstein et al., 2017; Vintimilla et al., 2019; Arce Rentería et al., 2020), whereas other studies have reported null associations or positive associations between hs-CRP and cognition (Alley et al., 2008; Hegazy et al., 2022). For example, a study of high functioning older adults showed that after controlling for potential cofounders, hs-CRP was not negatively associated with baseline cognitive function or rate of cognitive change (Alley et al., 2008). A more recent observational study of 111,242 individuals from the general population showed that low plasma levels of hs-CRP were associated with higher risk of AD (Hegazy et al., 2022). hs-CRP levels are generally quite variable and high levels can result from a wide variety of conditions from infections (Sproston and Ashworth, 2018) to cancer (Guo et al., 2013), as well as high levels of psychological stress (Johnson et al., 2013). Although statistically significant, the associations between hs-CRP and cognition were quite small and variability among hs-CRP was high. Sensitivity analyses removing individuals with high CRP values strengthened the positive relationships between CRP and cognition in our sample, suggesting that positive relationships between CRP and cognition are less likely when CRP levels are above clinical cut-offs.

### 4.3. Weak/non-consistent associations between CVRF biomarkers and cognition

Negative associations were observed between triglycerides, homocysteine, and several cognitive domains, however these relationships were relatively weak and inconsistent. High triglycerides is associated with cognitive impairment in large longitudinal studies (Kalmijn et al., 2000; Power et al., 2018), but several cross-sectional studies have shown no correlation, or even a reduction in dementia risk (Yin et al., 2012; Zhao et al., 2019; Buyo et al., 2020). Associations observed between homocysteine and executive function are consistent with the literature. A systematic review of 111 studies concluded that there is a negative but non-significant association between cognition and plasma homocysteine levels in individuals with and without cognitive impairment, with executive function being a highly affected domain (Setién-Suero et al., 2016). The relationship between homocysteine and cognition was attenuated in several studies after accounting for covariates such as white matter lesions.

Mixed results were observed with protein carbonyls showing some positive and negative associations with different cognitive domains. Higher protein carbonyl values were associated with better executive function overall. When stratified by sex, protein carbonyls were positively associated with verbal episodic memory at baseline in males, but negatively associated with processing speed in females. Protein carbonylation reflects oxidative damage (Dalle-Donne et al., 2003), but the link between protein carbonyls and cognition remain largely underexplored in older adults. A study investigating correlations between serum carbonyl compound profiles and cognitive impairment found that patients with subclinical carotid atherosclerosis had worse cognition and significant differences in carbonyl profile compared to healthy controls (Wu et al., 2018). Of 14 identified compounds, six were correlated with cognitive performance, showing both negative and positive correlations.

### 4.4. Sex differences

The relationships between CVRF biomarkers and cognition were more consistent in females relative to males. It has been suggested that the increased prevalence of cognitive impairment in females may be explained in part by sex-related differences in CVRFs (Volgman et al., 2019). A longitudinal study of 1857 participants (aged 50–69) found that cardiovascular conditions such as coronary artery disease, arrhythmias, and congestive heart failure were strongly associated with global cognitive decline, and CVRFs such as diabetes and dyslipidemia were associated with declines in language scores in females, but not males (Huo et al., 2022). Results stemming from the United Kindom Biobank study (*n* = 502,226, mean age = 56.5 years) suggested that smoking, diabetes, and adiposity, factors which promote oxidative damage, were associated with a greater risk of dementia in both males and females equally (Gong et al., 2021). Closely aligned with the current results, a recent study of 4,217 participants found that high levels of hs-CRP were associated with cognitive dysfunction in males only; whereas females with lower hs-CRP showed the steepest declines in cognitive functioning (Noss et al., 2022). There is also past research showing that HDL-C has a protective effect on cognition in males, but a slight adverse effect in females (Liu et al., 2020).

Although sex interactions were not observed in the relationship between HDL-C and cognition, sex stratified analyses showed stronger and more consistent associations in females. The relationships between glucose, hs-CRP and cognition were also observed primarily in females. Removing individuals with high glucose and hs-CRP values from the analysis revealed more robust associations between glucose and cognition in the remaining males, attenuating sex differences. This suggests that at higher levels of cardiovascular and cardiometabolic risk, CVRF’s may be negatively affecting cognition in males more than females. Based on the extant literature and current findings, sex differences in the association between CVRF biomarkers and cognition are likely; however a better understanding of the underlying biological mechanisms explaining these relationships and its potential implications is required.

### 4.5. Strengths and limitations

The strengths of this study include the evaluation of fasting blood and saliva concentrations of several CVRF biomarkers and the assessment of four cognitive domains using a battery of validated neuropsychological tests. In addition, our analyses evaluated sex differences and accounted for potential socio-demographic, lifestyle, and comorbidity confounders known to impact brain health. Lastly, the inclusion of cognitively healthy individuals whose CVRF markers lie within healthy ranges is pertinent to understanding the relationships between these markers and cognition prior to the presence of cardiovascular disease. The results from this study may help to inform practitioners about the utility of CVRFs in predicting cognition in individuals following routine blood measurement.

Study findings must also be interpreted with caution considering several study limitations. First, the observational analyses in this study precludes the determination of causality and does not account for residual confounding. Second, a duration of 2-year of follow-up limits the ability to observe cognitive decline and perform longitudinal analyses. Lastly, the sample size was compromised due to missing covariate data and participant attrition, which minimized statistical power of the current analyses. Although reasons for discontinuing participation is unknown, it may reflect worsening of metabolic health and cognition, and even death at follow-up. While this does not impact baseline associations, it introduces a bias for healthier participants at follow-up assessment. Selection bias is frequent during longitudinal studies (Mayeda et al., 2016), which may result in spurious risk or protective effects. This may have dampened estimated associations between CVRF biomarkers and cognition at follow-up, and may partially explain the unexpected associations pertaining to fasting glucose and CRP. Furthermore, recruiting within a nutritional study may favor the inclusion of more health-conscious individuals, however participants were recruited at baseline from random samples of Medicare lists. Thus, our results are to be generalized to cognitively healthy older adults, not the general older population.

Lastly, the large number of analyses run on the dataset increases the chance of false positive rates when determining statistical significance of associations. While type 3 test *p* values were used to indicate overall associations, as well as time and sex interactions, the current study cannot assert that these associations represent true relationships in the population. These tests and p-values were used to guide comparisons of relationships rather than hypothesis testing.

### 4.6. Conclusion

High-density lipoprotein-cholesterol showed the most robust associations with cognition in our sample. In addition, the relationships between CVRF biomarkers and cognition observed in our study were more consistent in females compared to males. Contrary to our expectations, the majority of CVRF biomarkers were not negatively associated with cognition, and it may be the case that prior to reaching levels associated with cardiovascular risk, these biomarkers contribute to healthy metabolic and neural function. Future studies with more participants and longer durations suitable for longitudinal analysis would help to provide an understanding of the long-term effects of CVRF biomarkers, investigate potential sex differences, and gain a better understanding of how the relationships between CVRF biomarkers and cognition differs between healthy individuals and those with greater cardiometabolic risk.

## Data availability statement

The original contributions presented in the study are included in the article/supplementary materials, further inquiries can be directed to the corresponding author/s.

## Ethics statement

The studies involving humans were approved by The NuAge study and the embedded NutCog study protocols were approved by the Research Ethics Boards of the Institut universitaire de gériatrie de Montréal and the Institut universitaire de gériatrie de Sherbrooke (Quebec, Canada). The NuAge Database and Biobank have been approved by the Research Ethic Board of the CIUSSS de l’Estrie–CHUS (Quebec, Canada). The studies were conducted in accordance with the local legislation and institutional requirements. The participants provided their written informed consent to participate in this study.

## Author contributions

NK: Writing – original draft, Validation. P-HC: Formal Analysis, Writing – review & editing. SB: Conceptualization, Data curation, Investigation, Methodology, Writing – review & editing. AF: Supervision, Writing – review & editing. PG: Data curation, Investigation, Supervision, Writing – review & editing. CG: Supervision, Writing – review & editing. M-JK: Methodology, Supervision, Writing – review & editing. JM: Methodology, Writing – review & editing. NP: Data curation, Investigation, Methodology, Supervision, Writing – review & editing. DL: Methodology, Supervision, Writing – review & editing. GF: Conceptualization, Funding acquisition, Investigation, Methodology, Project administration, Supervision, Writing – review & editing.
