# Supplementary material for: Associations between circulating cardiovascular disease risk factors and cognitive performance in cognitively healthy older adults from the NuAge study
Source: Front Aging Neurosci. 2023 Nov 10;15:1274794. doi: 10.3389/fnagi.2023.1274794 (PMC10668121; doi:10.3389/fnagi.2023.1274794)
Supplement: Supplementary file 1 [file Data_Sheet_1.docx]

**Appendix A**

**Table A1.** CVRF biomarker values and cognition at baseline in completers and non-completers

|  | **Completers**  **(n = 315)** | **Non-completers (n = 71)** | **p-value** |
| --- | --- | --- | --- |
| CVRF Biomarkers  Total cholesterol (mmol/L) | 5.10 ± 0.93 | 5.06 ± 0.95 | 0.762 |
| HDL-C (mmol/L) | 1.34 ± 0.37 | 1.31 ± 0.37 | 0.479 |
| LDL-C (mmol/L) | 3.05 ± 0.81 | 2.97 ± 0.82 | 0.437 |
| Triglycerides (mmol/L) | 1.55 ± 0.72 | 1.73 ± 0.84 | 0.071 |
| Fasting glucose (mmol/L) | 5.49 ± 0.95 | 5.94 ± 1.78 | 0.048 |
| Insulin (pmol/L) | 19.91 ± 16.91 | 18.38 ± 7.58 | 0.251 |
| HOMA-IR | 4.95 ± 4.21 | 5.03 ± 2.96 | 0.862 |
| Homocysteine (mcmol/L) | 12.72 ± 4.09 | 13.61 ± 4.33 | 0.113 |
| hs-CRP (mg/L) | 2.54 ± 3.05 | 3.59 ± 4.61 | 0.103 |
| Cortisol (mcg/dL) | 3.20 ± 7.17 | 3.13 ± 4.10 | 0.915 |
| Protein carbonyls (nmol/mg) | 1.99 ± 1.31 | 2.26 ± 1.39 | 0.137 |
| Cognitive Domains |  |  |  |
| Verbal episodic memory (Z-score) | 0.44 ± 3.33 | -1.94 ± 3.64 | <0.001 |
| Non-verbal episodic memory (Z-score) | 0.18 ±1.89 | -0.79 ± 2.01 | <0.001 |
| Executive function (Z-score) | 0.30 ± 2.42 | -1.34 ± 3.08 | <0.001 |
| Processing speed (Z-score) | 0.10 ± 1.43 | -0.44 ± 1.90 | 0.025 |

*Note*: Means ± standard deviation. CVRF = cardiovascular risk factor, HDL-C = high density lipoprotein-cholesterol; LDL-C = low density lipoprotein-cholesterol; HOMA-IR = Homeostatic Model Assessment for Insulin Resistance; hs-CRP = high sensitivity C-reactive protein.

**Table A2.** Associations between CVRF biomarkers and cognition at baseline and follow-up in basic models

|  | **Whole sample** | |
| --- | --- | --- |
|  | ***β* (95% CI)** | ***β* (95% CI)** |
|  | Verbal Episodic Memory | Non-Verbal Episodic Memory |
| Total cholesterol^1^ | **0.61 (0.24; 0.98)** | 0.01 (-0.19; 0.22) |
| Total cholesterol^2^ | **0.42 (0.08; 0.77) *** | -0.06 (-0.26; 0.13) |
| HDL-C^1^ | **2.48 (1.57; 3.40)** | **0.63 (0.11; 1.14)** |
| HDL-C^2^ | **1.00 (0.05; 1.94) *^** | -0.23 (-0.76; 0.31) ^ |
| LDL-C^1^ | 0.41 (-0.02; 0.83) | -0.05 (-0.29; 0.19) |
| LDL-C^2^ | 0.39 (-0.02; 0.80) * | 0.02 (-0.21; 0.24) |
| Triglycerides^1^ | -0.32 (-0.79; 0.15) | -0.17 (-0.43; 0.09) |
| Triglycerides^2^ | 0.04 (-0.42; 0.50) | -0.18 (-0.43; 0.08) * |
| Glucose^1^ | -0.13 (-0.42; 0.17) | -0.12 (-0.29; 0.04) |
| Glucose^2^ | -0.05 (-0.43; 0.33) | -0.04 (-0.25; 0.17) |
| Insulin^1^ | -0.01 (-0.03; 0.01) | 0.00 (-0.01; 0.02) |
| Insulin^2^ | -0.00 (-0.03; 0.02) | -0.00 (-0.01; 0.01) |
| HOMA-IR^1^ | -0.06 (-0.15; 0.03) | 0.00 (-0.04; 0.05) |
| HOMA-IR^2^ | -0.02 (-0.10; 0.07) | -0.01 (-0.05; 0.04) |
| Homocysteine^1^ | **-0.12 (-0.20; -0.03)** | -0.02 (-0.07; 0.02) |
| Homocysteine^2^ | -0.05 (-0.14; 0.03) * | -0.00 (-0.05; 0.05) |
| hs-CRP^1^ | -0.02 (-0.13; 0.09) | 0.01 (-0.05; 0.07) |
| hs-CRP^2^ | -0.01 (-0.04; 0.03) | -0.01 (-0.03; 0.01) |
| Cortisol^1^ | 0.02 (-0.03; 0.08) | 0.01 (-0.02; 0.04) |
| Cortisol^2^ | 0.01 (-0.00; 0.03) | 0.01 (-0.00; 0.02) |
| Protein carbonyls^1^ | 0.23 (-0.03; 0.50) | -0.05 (-0.20; 0.09) |
| Protein carbonyls^2^ | -0.18 (-0.48; 0.13) ^ | -0.03 (-0.20; 0.14) |
|  |  |  |
|  | Executive Function | Processing Speed |
| Total cholesterol^1^ | 0.29 (-0.02; 0.59) | **0.19 (0.02; 0.36)** |
| Total cholesterol^2^ | 0.26 (-0.02; 0.54) * | 0.07 (-0.09; 0.23) * |
| HDL-C^1^ | **1.01 (0.26; 1.76)** | 0.05 (-0.38; 0.47) |
| HDL-C^2^ | **1.07 (0.30; 1.84) *** | -0.01 (-0.45; 0.43) |
| LDL-C^1^ | 0.26 (-0.09; 0.61) | **0.22 (0.02; 0.41)** |
| LDL-C^2^ | **0.35 (0.02; 0.67) *** | 0.12 (-0.07; 0.30) * |
| Triglycerides^1^ | -0.26 (-0.64; 0.12) | 0.05 (-0.16; 0.27) |
| Triglycerides^2^ | **-0.52 (-0.89; -0.15) *** | -0.05 (-0.26; 0.16) |
| Glucose^1^ | -0.19 (-0.43; 0.05) | -0.13 (-0.27; 0.00) |
| Glucose^2^ | -0.13 (-0.44; 0.17) * | -0.10 (-0.27; 0.07) * |
| Insulin^1^ | -0.00 (-0.02; 0.02) | -0.00 (-0.01; 0.01) |
| Insulin^2^ | 0.00 (-0.01; 0.02) | 0.00 (-0.01; 0.01) |
| HOMA-IR^1^ | -0.03 (-0.10; 0.04) | -0.02 (-0.06; 0.02) |
| HOMA-IR^2^ | -0.01 (-0.07; 0.06) | 0.01 (-0.03; 0.04) |
| Homocysteine^1^ | **-0.12 (-0.19; -0.06)** | -0.03 (-0.06; 0.01) |
| Homocysteine^2^ | **-0.10 (-0.16; -0.03) *** | -0.03 (-0.07; 0.01) * |
| hs-CRP^1^ | 0.03 (-0.06; 0.12) | 0.01 (-0.04; 0.06) |
| hs-CRP^2^ | 0.02 (-0.01; 0.04) | 0.01 (-0.00; 0.28) |
| Cortisol^1^ | 0.03 (-0.01; 0.07) | -0.00 (-0.02; 0.02) |
| Cortisol^2^ | 0.01 (-0.00; 0.02) * | -0.00 (-0.01; 0.01) |
| Protein carbonyls^1^ | 0.17 (-0.04; 0.38) | -0.01 (-0.10; 0.13) |
| Protein carbonyls^2^ | 0.22 (-0.02; 0.46) * | 0.01 (-0.13; 0.14) |

*Note*: HDL-C = high density lipoprotein cholesterol; ^1^ = baseline; ^2^ = follow-up; LDL-C = low density lipoprotein cholesterol; HOMA-IR = Homeostatic model assessment for insulin resistance; hs-CRP = high sensitivity C-reactive protein.

* = overall association (type 3 test p < .10); ^ = time interaction (p < .10). **Bolded** data denote associations with confidence intervals that do not include zero.

Models are adjusted for time and CVRF by time interaction.

**Table A3.** Sensitivity analyses removing individuals with high glucose and CRP levels from final models

|  | **Whole sample^a^** | **Males^b^** | **Females^b^** |
| --- | --- | --- | --- |
|  | ***β* (95% CI)** | ***β* (95% CI)** | ***β* (95% CI)** |
| Verbal Episodic Memory |  | | |
| HDL-C^1^ | **2.14 (1.14; 3.13)** | **2.14 (0.33; 3.95)** | **2.17 (0.99; 3.35)** |
| HDL-C^2^ | 0.39 (-0.64; 1.41) * ^ | 0.05 (-1.81; 1.92) ^ | 0.31 (-0.91; 1.53) * ^ |
| Glucose^1^ | **0.58 (0.04; 1.12)** | 0.46 (-0.39; 1.31) | **0.71 (0.00; 1.41)** |
| Glucose^2^ | **0.66 (0.14; 1.18)** * | 0.42 (-0.36; 1.20) | **0.82 (0.11; 1.54) *** |
| Executive Function |  | | |
| HDL-C^1^ | 0.80 (-0.04; 1.63) | -0.03 (-1.44; 1.37) | **1.42 (0.33; 2.51)** |
| HDL-C^2^ | 0.69 (-0.12; 1.50) ***** | 0.21 (-1.16; 1.59) | **1.32 (0.29; 2.36) *** |
| hs-CRP^1^ | 0.07 (-0.07; 0.21) | -0.06 (-0.26; 0.15) | 0.13 (-0.06; 0.33) |
| hs-CRP^2^ | 0.15 (-0.01; 0.31) ***** | 0.08 (-0.17; 0.33) | 0.21 (-0.01; 0.43) ***** |
| Processing Speed |  | | |
| hs-CRP^1^ | 0.04 (-0.01; 0.10) | 0.02 (-0.10; 0.13) | 0.11 (-0.01; 0.23) |
| hs-CRP^2^ | 0.03 (-0.03; 0.09) ***** | **0.16 (0.01; 0.31) *** | -0.04 (-0.18; 0.09) |

*Note*: CVRF = cardiovascular risk factor, HDL-C = high density lipoprotein-cholesterol; ^1^ = baseline; ^2^ = follow-up; hs-CRP = high sensitivity; C-reactive protein.

* = overall association (type 3 test p < .10); ^ = time interaction (p < .10). **Bolded** data denote associations with confidence intervals that do not include zero.

^a =^ Models are adjusted for time; CVRF by time interaction; age; sex; CVRF by sex interaction; education; BMI; diet quality (C-HEI); physical activity (PASE); smoking status; hypertension; and depressive symptoms (GDS).

^b =^ Models are adjusted for time; CVRF by time interaction; age; education; BMI; diet quality (C-HEI); physical activity (PASE); smoking status; hypertension; and depressive symptoms (GDS).

**Table A4.** Multivariate analysis showing associations between CVRF biomarkers and executive function performance before removing homocysteine and protein carbonyls as predictor variables.

|  | ***β* (95% CI)** |
| --- | --- |
| HDL-C^1^ | **0.57 (0.06; 1.08)** |
| HDL-C^2^ | **0.55 (0.07; 1.03) *** |
| hs-CRP^1^ | **0.06 (0.00; 0.12)** |
| hs-CRP^2^ | **0.06 (0.01; 0.11) *** † |
| Homocysteine^1^ | -0.02 (-0.08; 0.03) |
| Homocysteine^2^ | -0.02 (-0.07; 0.03) |
| Protein carbonyls^1^ | 0.06 (-0.08; 0.20) |
| Protein carbonyls^2^ | 0.03 (-0.12; 0.18) |

*Note*: CVRF = cardiovascular risk factor; HDL-C = high density lipoprotein-cholesterol; ^1^ = baseline; ^2^ = follow-up; hs-CRP = high sensitivity C-reactive protein.

* = overall association (type 3 test p < .10); † = sex interaction (p <.10). **Bolded** data denote associations with confidence intervals that do not include zero.

Models are adjusted for time, CVRF by time interaction, age, sex, CVRF by sex interaction, education, diet quality (C-HEI), physical activity (PASE), BMI, hypertension, depressive symptoms (GDS), and smoking status.
